# Supplementary material for: Reduced OPA1, Mitochondrial Fragmentation and Increased Susceptibility to Apoptosis in Granular Corneal Dystrophy Type 2 Corneal Fibroblasts
Source: Genes (Basel). 2023 Feb 24;14(3):566. doi: 10.3390/genes14030566 (PMC10048436; doi:10.3390/genes14030566)
Supplement: Supplementary file 1 [file genes-14-00566-s001.zip › genes-2193969-supplementary.pdf]

Supplementary Materials

Supplementary Table S1. Antibodies used in this study

Antibodies	Provider	Catalog Number	Experiment	Results (Figure)
Anti-OPA1	BD Transduction Laboratoris, Inc. USA	612606	WB*	Figure 1, 3,4 Sup.1
Anti-MFN1	NOVUS Biological, Littleton, CO	NBP1-71775	WB	Figure 1, 3 Sup.1
Anti-MFN2	Abcam	Ab 56889	WB	Figure 1, 3 Sup.1
Anti-pDRP1	Cell Signaling, Beverly, MA	#4867	WB	Figure 1, 3 Sup.1
Anti-DRP1	Cell Signaling, Beverly, MA	#8570	WB	Figure 1, 3 Sup.1
Anti-FIS1	IMGENEX, San Diego, CA	IMG5113A	WB	Figure 1, 3 Sup.1
Anti-TGFBIp	R&D Systems, Minneapolis, MN, USA.	AF2935	WB	Figure1
Anti-PARP1	Cell Signaling Technology Beverly, MA, USA.	#9532S	WB	Figure 4
Anti- Cleaved PARP1	Cell Signaling Technology Beverly, MA, USA.	#3868S	WB	Figure 4
Anti-Caspase 3	Cell Signaling Technology Beverly, MA, USA.	#9662S	WB	Figure 4
Anti-Cleaved Caspase 3	Cell Signaling Technology Beverly, MA, USA.	#9661S	WB	Figure 4
Total OXPHOS complexes detection kit	Mitosciences Inc., Eugene, OR.	MS601	WB	Figure 5
Anti-NRF1	Cell Signaling Technology Beverly, MA, USA.	#8882	WB	Figure 6
Anti-NRF2	Abcam	Ab56889	WB	Figure 6
Anti-PGC1α	Cell Signaling Technology Beverly, MA, USA.	#2178	WB	Figure 6
Anti-DLP1	BD Transduction Laboratoris, Inc. USA	611112	WB	Sup.1
Anti-GAPDH	Merck Millipore, Billerica, MA, USA.	MAB374	WB	Figure 3 Sup.1
Anti-TOM20	Santa Cruz, CA, USA	SC11415	WB	Figure 5,6 Sup.1
Anti-TIM23	Abcam	Ab116329	WB	Figure 6
Anti-Histone H3	Calbiochem, San Diego, CA, USA.	Ab1791	WB	Figure 6
Anti-β-actin	Sigma-Aldrich, St. Louis, MO, USA.	A5441	WB	Figure 1,4 Sup.1
Anti-mouse IgG	Thermo Fisher Scientific	NCI1430	WB	
Anti-rabbit IgG	Thermo Fisher Scientific	NCI1460	WB	

WB*: Western blots
Sup: Supplementary data

Supplementary Table S2. Materials used in this study

Name	Provider	Catalog Number	Experiments	Results (Figure)
Carbonyl cyanide m-chlorophenylhydrazone (CCCP)	Sigma-Aldrich, St. Louis, MO, USA.	C2759	Mitochondrial toxicity assay	Figure 7,4
Mdivi-1 (3-(2,4-dichloro-5-methoxyphenyl)-2-sulfanyl-4(3H)-quinazolinone)	Sigma-Aldrich, St. Louis, MO, USA.	M0199	Mitochondrial toxicity assay	Figure 7
OPA1 shRNA (h) Lentiviral Particles	Santa Cruz, CA, USA	sc-106808-V	*OPA1* gene knockdown	Figure 3, 4
Dulbeco's Modified Eagle's Medium (DMEM)	Corning Inc., Corning, NY, USA	10-013-CV	Cell culture	
Fetal Bovine Serum	Corning Inc., Corning, NY, USA	35-015-CV	Cell culture	
Presto Blue	Invitrogen, Life Technologies, USA.	A13261	Cell proliferation assay	Figure 4
MitoTraker Red FM	Thermo Fisher Scientific	M22425	Mitochondrial staining	Figure 1
MitoTraker Green FM	Thermo Fisher Scientific	M7514	Mitochondrial staining	Figure 1

Suppl: Supplementary data

2. SUPPLEMENTARY METHODS

Supplementary method 1. Cell Viability Assay

In brief, corneal fibroblasts (5×10^3 cells/well) were plated into 96-well plates (Corning, Inc., Corning, NY) and incubated overnight. They were treated with different concentrations of CCCP, either alone or in combination with Mdivi-1, and incubated for 15 h. The Presto Blue reagent was added to the wells and the plates were incubated for 4 h, after which fluorescence was measured (540 nm excitation/590 nm emission). These experiments were performed in triplicates and data are expressed as mean ± SD. Student's t-test was used to determine statistical significance (*$p < 0.05$ and **$p < 0.01$ compared to control).

Supplementary method 2. Preparation of Cell Lysates and Western Blot Analysis

The supernatant was used to determine the total protein concentration using a bicinchoninic acid kit (Pierce). Total cellular proteins (~50 μg) were electrophoresed in NuPAGE™ 4-12 % Bis-Tris Protein Gels (Invitrogen: NP0335). Proteins were transferred to polyvinylidene difluoride membranes with the iBlot 2 Dry Blotting System (Thermo Fisher Scientific), blocked in 5 % dry milk in Tris-buffered saline containing Tween 20 (TBS-T; 0.02 mol/L Tris, 0.15 mol/L NaCl, pH 7.5, containing 0.1 % Tween 20) at room temperature for 1 hour, washed three times with TBS-T, and then incubated with primary antibodies (supplementary table 1) at 4°C overnight. After washing three times with TBS-T, the blots were incubated with secondary antibodies conjugated with horseradish peroxidase at room temperature for 1 h. Immunoreactive band intensities were quantified using a computer software (ImageJ software, version 1.37, Wayne Rasband, NIH), corrected by background subtraction, and normalized to the intensity of the corresponding β-actin or GAPDH (glyceraldehyde-3-phosphate dehydrogenase) protein bands.

Supplementary method 3. Quantification of mtDNA Copy Number and Deletion

Copy numbers of mtDNA or nuclear DNA (nDNA) were calculated from the threshold cycle and

standard curve. mtDNA deletion was assayed as described previously.[1] To screen the 4,977 base pair (bp) deletion in mtDNA, we used the DNA samples extracted from the cells as templates and two pairs of primers, as described previously.[1] The PCR products were electrophoresed on a 1.2% agarose gel. The deletion was indicated by the appearance of a 358-bp band. The mtDNA of WT cells was used as the control and yielded no or little PCR products under such conditions because of the large flanking region (>5 kb).[1] Relative amounts of mtDNA and nDNA copy numbers were compared. The ddCt (mtDNA to 18S) represents the mtDNA copy number in a cell.

Supplementary method 4. Mitochondrial Network Imaging by Fluorescence and Electron Microscopy

Briefly, cells were incubated these dyes and fluorescence was visualized using a Nikon Eclipse E800 microscope with a fluorescein filter and a water immersion objective (60×). All images were captured with equal exposure times. Fluorescent images were quantified for each sample by averaging the mean intensity fluorescence of 5 random cells in 3 different fields using Nikon Nis Elements software. Conventional transmission electron microscopic (EM) analysis was performed as described previously.[2] Briefly, cultured corneal fibroblasts were fixed with overnight, dehydrated, processed, and evaluated under a transmission electron microscope (JEM1200 EX2; JEOL Ltd., Tokyo, Japan).

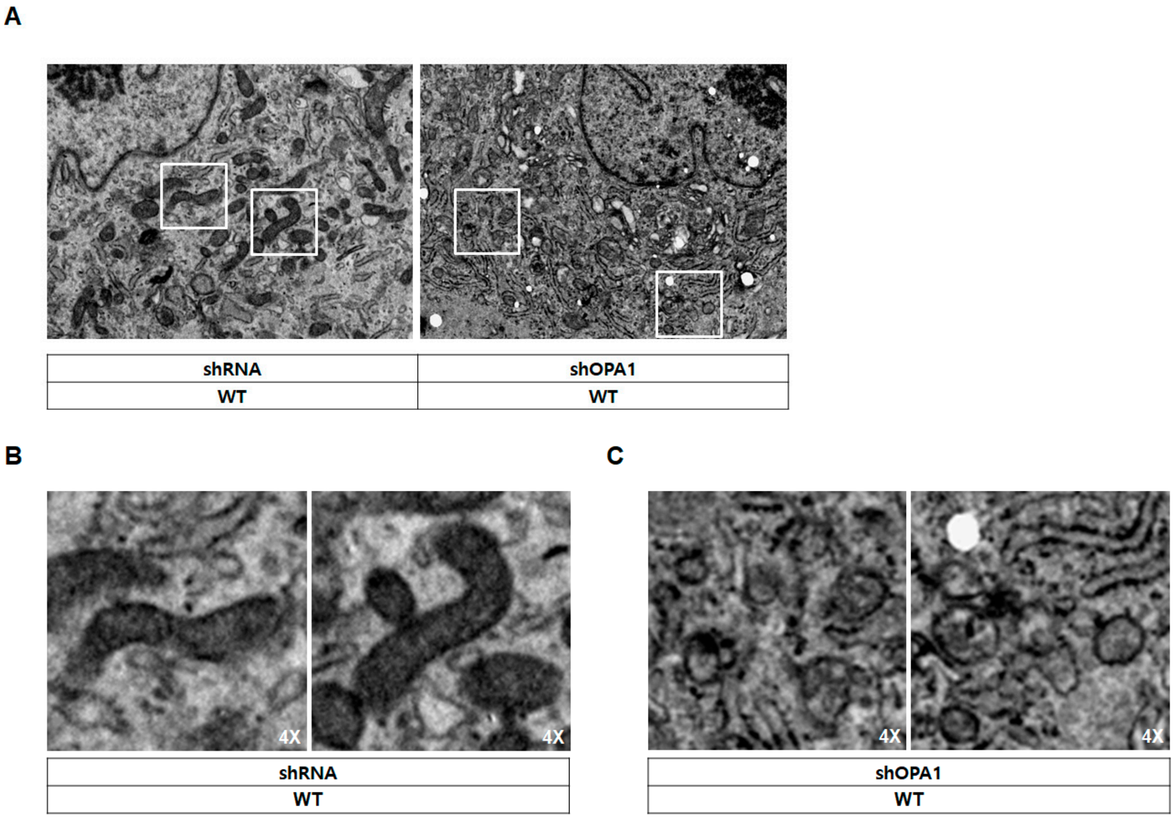

Figure S1: Mitochondrial ultrastructural abnormalities in scrambled shRNA and OPA1-specific shRNA-expressing in corneal fibroblasts.

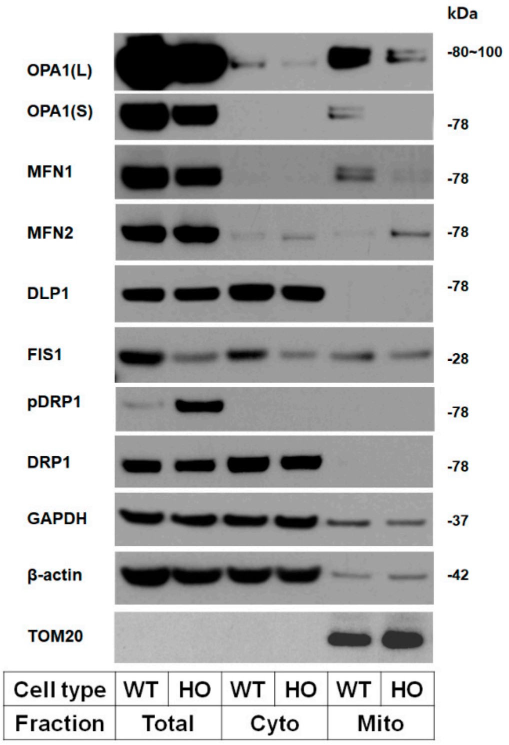

Figure S2: Mitochondrial fusion/fission-related protein levels in total, cytosol, and mitochondrial fraction of WT and GCD2 HO corneal fibroblasts.

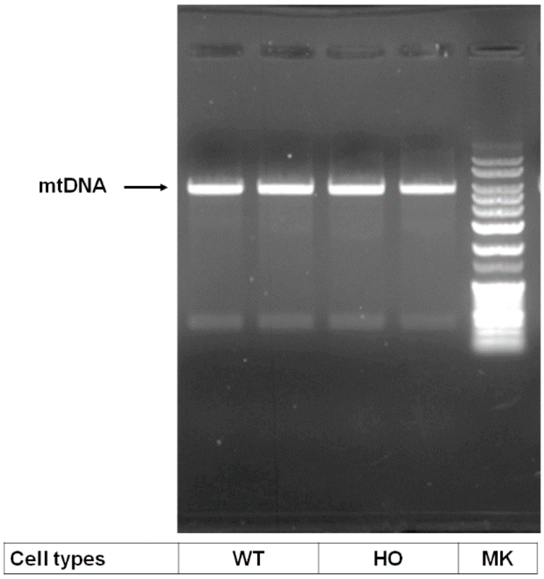

Figure S3. Detection of the Mitochondrial DNA Deletion.

References

1. Malik, A.N.; Shahni, R.; Rodriguez-de-Ledesma, A.; Laftah, A.; Cunningham, P. Mitochondrial DNA as a non-invasive biomarker: Accurate quantification using real time quantitative PCR without co-amplification of pseudogenes and dilution bias. *Biochem. Biophys Res. Commun.* **2011**, *412*, 1–7. https://doi.org/10.1016/j.bbrc.2011.06.067.

2. Kim TI, Kim H, Lee DJ, Choi SI, Kang SW, Kim EK. Altered mitochondrial function in type 2 granular corneal dystrophy. *The American journal of pathology*. 2011;179:684-692.
